# Sub-5 nm porous nanocrystals: interfacial site-directed growth on graphene for efficient biocatalysis†
†Electronic supplementary information (ESI) available. See DOI: 10.1039/c5sc00819k


**DOI:** 10.1039/c5sc00819k

**Published:** 2015-04-14

**Authors:** Biao Kong, Xiaotian Sun, Cordelia Selomulya, Jing Tang, Gengfeng Zheng, Yingqing Wang, Dongyuan Zhao

**Affiliations:** a Department of Chemistry , Collaborative Innovation Center of Chemistry for Energy Materials (iChEM) , Laboratory of Advanced Materials , Shanghai Key Laboratory of Molecular Catalysis and Innovative Materials , Fudan University , Shanghai 200433 , P. R. China . Email: dyzhao@fudan.edu.cn; b Department of Chemical Engineering , Monash University , Clayton , VIC 3800 , Australia; c Department of Cardiothoracic Surgery , Huashan Hospital of Fudan University , Shanghai 200040 , P. R. China . Email: wangyiqing@huashan.org.cn

## Abstract

The direct production of macromolecular scale (sub-5 nm) porous nanocrystals with high surface area has been a considerable challenge over the past two decades. Here we report an interfacial site-directed capping agent-free growth method to directly produce porous ultrasmall (sub-5 nm), fully crystalline, macromolecular scale nanocrystals. The porous sub-5 nm Prussian blue nanocrystals exhibit uniform sizes (∼4 ± 1 nm), high surface area (∼855 m^2^ g^–1^), fast electron transfer (rate constant of ∼9.73 s^–1^), and outstanding sustained catalytic activity (more than 450 days). The nanocrystal-based biointerfaces enable unprecedented sub-nanomolar level recognition of hydrogen peroxide (∼0.5 nM limit of detection). This method also paves the way towards the creation of ultrasmall porous nanocrystals for efficient biocatalysis.

## Abstract 2

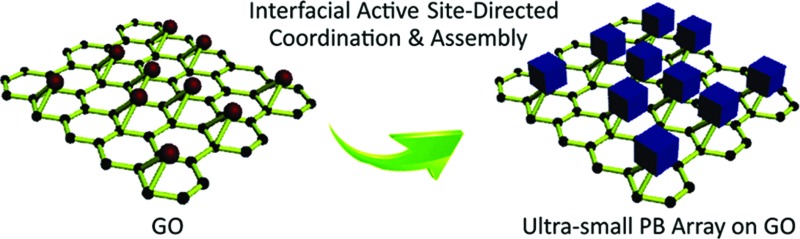
An interfacial site-directed, capping-agent-free growth method for direct production of macromolecular scale (sub-5 nm) porous nanocrystals that are fully crystalline with a high surface area were developed for efficient biocatalysis.

## Introduction

**Fig. 1:**
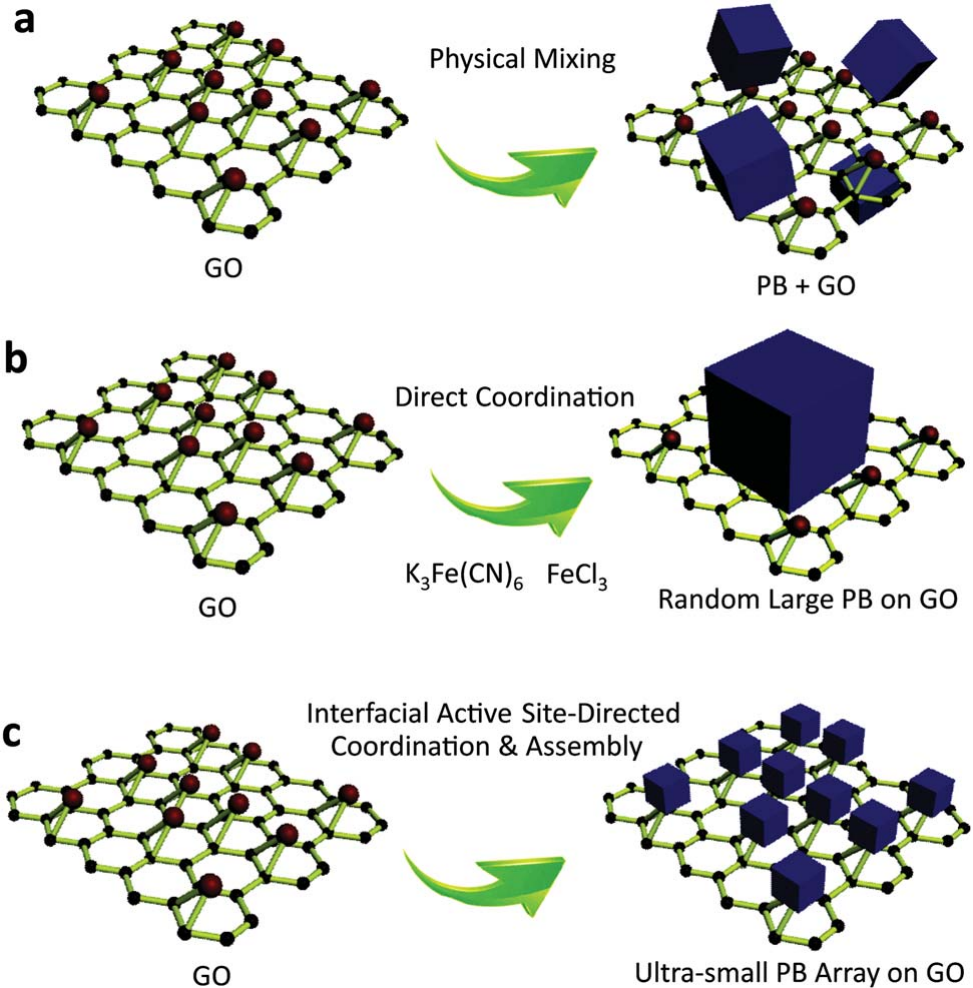
Comparison of the classic physical mixing, direct coordination, and interfacial site-directed growth approaches for sub-5 nm porous Prussian blue nanocrystals. (a) Mixture of Prussian blue and graphene oxide resulting from the physical mixing method. (b) Random large Prussian blue growth on graphene oxide by the direct coordination method. (c) Ultrasmall porous Prussian blue assembly arrays on graphene resulting from the interfacial site-directed growth method.

Recent advances in nanotechnology have given rise to a new class of ultrasmall, sub-5 nm nanocrystals,^1^,^2^ such as quantum dots,^3^ carbon nanodots,^4^,^5^ graphene nanodots,^6^ and metal nanoclusters.^7^ These sub-5 nm nanocrystals, composed of a few to several hundred atoms, are of significant interest because they provide the missing link between atomic and nanoparticle behavior.^8^,^9^ Their ultrasmall sizes are comparable to the Fermi wavelength of electrons,^10^ resulting in unusual optical, electrical and chemical properties that differ markedly from larger nanocrystals.^11^ Porous materials are of scientific and technological interest with broad applications in catalysis,^12^ gas separation,^13^ chemical sensing,^14^ and optical devices^15^,^16^ due to their large and accessible specific surface areas, tunable and uniform pore sizes, and diverse properties.^17^,^18^ Their ability to perform a desired function is sensitive to slight variations in the distribution of the size and volume of void spaces in the porous framework.^19^,^20^ The rational design and fabrication of integrated ultrasmall (sub-5 nm) porous nanocrystals can offer properties of both small size and accessible porosity, leading to a series of tunable functional platforms.^21^ Currently, the fabrication of ultrasmall nanocrystals is typically accomplished by solvothermal processes,^22^ cothermolysis methods,^23^ simultaneous precipitation,^24^ thermal decomposition,^25^ multiphase mass transfer,^26^ microwave irradiation,^27^,^28^ biomolecule capping,^29^ and photoreduction^30^ strategies. In most of the existing approaches for ultrasmall nanocrystals, the use of suitable reagents capable of stabilizing the nanocrystals and preventing their aggregation is usually necessary. For porous materials, however, the self-assembly of sub-5 nm pore structures is sensitive to potential changes in the assembly conditions,^31^ especially in the presence of exogenous capping reagents,^32^ which largely limits the possibility of synthesizing sub-5 nm pore materials using existing approaches. Until now, the synthesis of macromolecular scale (sub-5 nm) porous nanocrystals has remained a challenge (Fig. 1).

Herein, taking the first synthetic coordination compound, Prussian blue (PB, ferric hexacyanoferrate) as an example, we present atomic level, site-directed, capping agent-free growth of porous sub-5 nm Prussian blue nanocrystals on graphene. As a proof-of-concept, the sub-5 nm porous Prussian blue nanocrystals show ultrasmall size (<5 nm), narrow size distribution (4 nm ± 1.5 nm), high surface area (∼855 m^2^ g^–1^), ultrafast electron transfer (rate constant of ∼9.73 s^–1^), and persistent catalytic activity (more than 450 days), leading to greatly enhanced catalytic performance (∼85-fold increase). Specifically, the nanocrystal–graphene heterointerface demonstrates an unprecedented sub-nanomolar level (∼0.5 nM limit of detection) for capturing and recognising hydrogen peroxide (H_2_O_2_) that has never been shown with traditional biointerfaces to date. This approach adds to the synthetic toolbox for nanocrystals and mesoporous materials, creating ultrasmall porous nanostructures by interfacial site-directed growth that have previously been impossible to achieve by traditional approaches. The results should provide an improved understanding of the synergistic effect resulting from the integration of small size and accessible porosity, which is important for developing heterointerfaces for catalytic applications.

## Results

### Fabrication of sub-5 nm porous Prussian blue nanocrystals

**Fig. 2:**
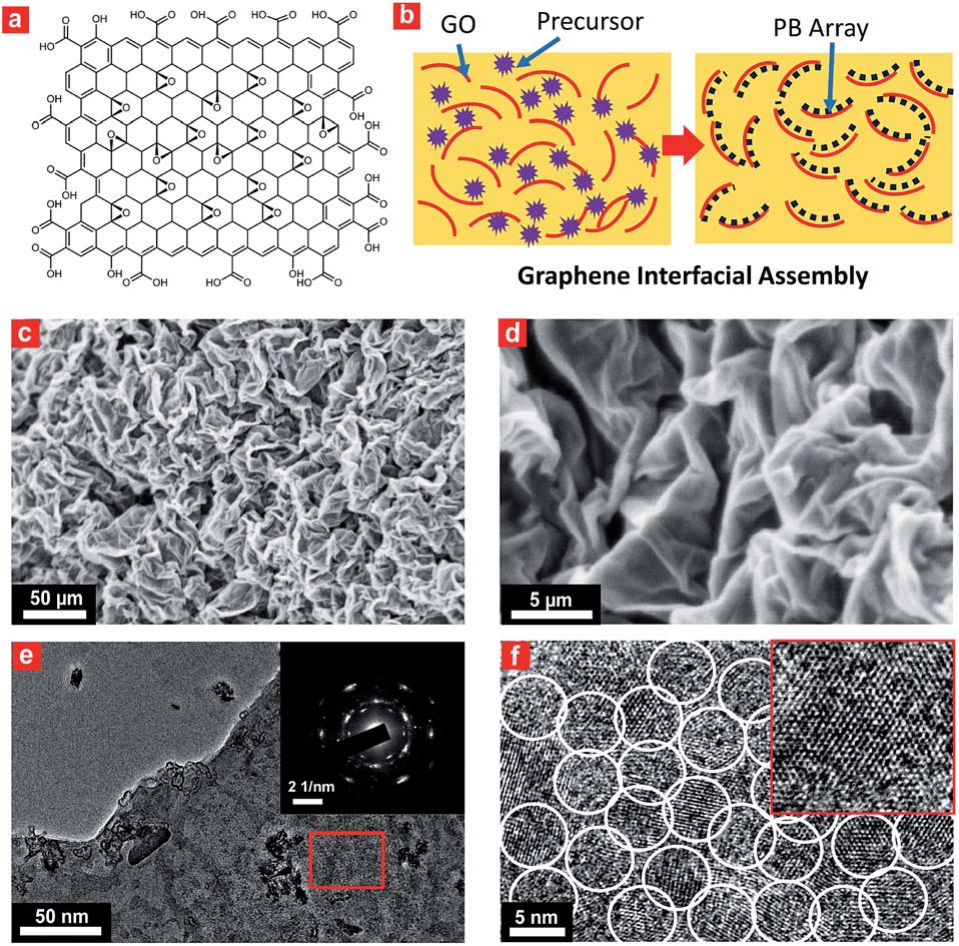
Characterization of the ultrasmall porous Prussian blue nanocrystals on graphene. (a) A typical amphiphilic graphene oxide structural model with hydrophobic sites (π domains) and hydrophilic sites (–COOH groups). (b) Schematic illustration showing the interfacial site-directed growth of ultrasmall porous Prussian blue nanocrystals on graphene oxide. Precursors of the Prussian blue nanoclusters were first captured by the active hydrophilic sites and then interacted with the graphene surface for synchronous reduction and growth of Prussian blue nanocrystals. (c and d) SEM, (e) TEM, and (f) HRTEM images of the obtained ultrasmall porous Prussian blue–graphene composite structure. Inset in (f): HRTEM image of a single ultrasmall porous Prussian blue nanocrystal.

Interfacial site-directed growth of sub-5 nm Prussian blue nanocrystals was achieved by controlled hydrolysis, coordination and assembly of molecular precursors on the graphene interface without structure-directing surfactants or capping agents (Fig. 2). Graphene oxide is hydrophilic and can be dispersed in water, mainly due to the number of hydrophilic oxygenated functional groups (Fig. 2a).^33^ Graphene oxide (GO) was synthesized using a modified Hummers' method and dispersed in water by sonication (4.0 mg mL^–1^).^34^ The precursor of Prussian blue, K_3_[Fe(CN)_6_]·3H_2_O, was slowly added to the GO dispersion to form a stable aqueous suspension for pre-interaction of the precursor of Prussian blue with interfacial reactive sites on the GO sheets, followed by hydrothermal treatment to give Prussian blue-anchored graphene nanosheets (Fig. 2b). In this way, sub-5 nm porous Prussian blue nanocrystals nucleate and grow on the graphene surface with simultaneous reduction of oxygenated functional groups on the graphene surface. Representative scanning electron microscopy (SEM) images (Fig. 2c and d and S1†) of the Prussian blue–graphene nanosheets show randomly dispersed, crumpled sheets closely associated with each other and forming a highly exfoliated bundle. The transmission electron microscopy (TEM) characterization further validates the successful growth of Prussian blue nanocrystals on the GO sheets. The inset shows the electron diffraction pattern of the as-prepared Prussian blue–graphene, indicating excellent crystallization of the nanosheets (Fig. 2e).^35^ The representative high-resolution transmission electron microscopy (HRTEM) image (Fig. 2f and S2†) shows that many uniform nanocrystals of Prussian blue with average sizes of ∼4.5 nm are homogeneously anchored to the surface of the graphene sheets. The HRTEM image shows a typical single Prussian blue nanocrystal with a highly crystalline texture (inset, Fig. 2f). The electron dispersive X-ray (EDX) spectra reveal the presence of the Fe component in the Prussian blue–graphene sheets (Fig. S3a†).^36^ X-ray photoelectron spectroscopy (XPS) revealed two peaks at ∼725 and 711 eV, which were assigned to Fe 2p1/2 and Fe 2p3/2, respectively (Fig. S3b†).^36^,^37^ The Prussian blue nanocrystal–graphene heterostructures were further examined by X-ray diffraction (XRD), which showed clear diffraction peaks of Prussian blue (Fig. S3c†).^36^ Brunauer–Emmett–Teller (BET) analysis showed a specific surface area of ∼855 m^2^ g^–1^ for the Prussian blue–graphene framework, together with hierarchical porous features.

### Fabrication of 3D porous Prussian blue nanocrystal-based hydrogel

**Fig. 3:**
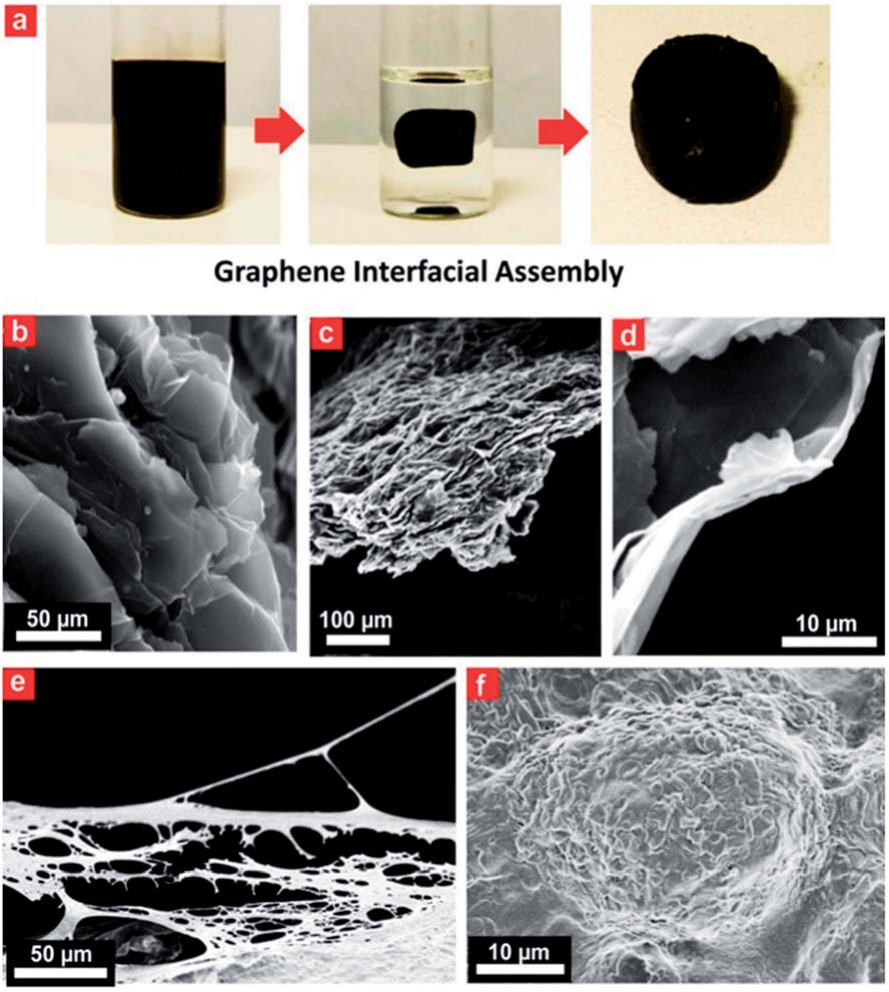
Ultrasmall Prussian blue nanocrystal hydrogel. (a) Optical photographs of the ultrasmall Prussian blue nanodot hydrogel during the formation process. (b and c) SEM and (d) enlarged SEM images of the obtained ultrasmall Prussian blue nanodot hydrogel. (e) SEM and (f) enlarged SEM images of the obtained ultrasmall Prussian blue nanodot hydrogel-based cell interface.

A porous Prussian blue–graphene-based 3D hydrogel could be obtained by hydrothermal assembly at 180 °C for 12 h (Fig. 3). The as-prepared hydrogel was directly dehydrated *via* a freeze-drying process to maintain the 3D monolithic architecture and then used as a biomonitoring interface. The final product from this process was a black monolithic hybrid aerogel composed of graphene networks and Prussian blue nanocrystals (Fig. 3a). TEM images reveal an interconnected, 3D porous Prussian blue–graphene framework with continuous macropores in the micrometer size range (Fig. 3b and c). Apart from the formation of 3D macropores (>50 μm) on the Prussian blue–graphene framework by stacking of Prussian blue–graphene sheets (Fig. 3b), a significant stacking of pores (∼10 μm) within the Prussian blue–graphene layers takes place (Fig. 3c and d).

### Electrochemical performance

Electrochemical interfaces enable fast, low-cost, real time and *in situ* probing of biosignals in living cells and organisms. The electrochemical performance of the porous Prussian blue–graphene heterostructure was investigated by cyclic voltammetry (CV). Pristine Ti foil was also measured under similar conditions for comparison. No obvious redox peaks except for the capacitive current were observed for the pristine Ti foil electrode, while the porous Prussian blue–graphene electrode displayed a pair of redox peaks at 0.18 and 0.23 V, corresponding to the reversible redox conversion of Prussian blue to Prussian white (Fig. S4a†). The electrochemical stability of the porous Prussian blue–graphene was also demonstrated by repeated CV measurements at a scan rate of 50 mV s^–1^. No clear difference was observed in either the current level or peak positions of the CV curves after 200 and 500 cycles, confirming the stability of the immobilized Prussian blue nanocrystals on graphene (Fig. S4b†). The scan rate-dependent voltammetry profile of the porous Prussian blue–graphene electrode in the range 50–1000 mV s^–1^ was then measured (Fig. S4c†). The anodic and cathodic peak potentials for direct electron transfer of Prussian blue are dependent on scan rate (Fig. S4c†). The anodic and cathodic peak potentials shift slightly in the positive and negative directions, respectively. Δ*E*_p_ increases with increasing scan rate, however, the value of *E*_1/2_ is independent of the scan rate. From the dependence of Δ*E*_p_ on the scan rate, the apparent heterogeneous electron transfer rate constant (*k*_s_) was calculated to be 9.73 ± 0.25 s^–1^ using a surface-controlled electrochemical method.^38^ This value of *k*_s_ is much higher than most of the H_2_O_2_ electrodes reported previously (Table S1†). It is well-known that the reduced form of Prussian blue has a high catalytic activity for H_2_O_2_ catalysis. Thus, the ability to use the Prussian blue–graphene electrode as an amperometric monitor for H_2_O_2_ was further investigated.^27^ The injection of H_2_O_2_ (5 mM in PBS, pH 6.0) led to a clear increase in current density (corresponding to H_2_O_2_ reduction), at a lower overpotential (–50 mV *versus* Ag/AgCl) (Fig. S4d†). The electrochemical response for hydrogen peroxide reduction depends on the activity of Prussian blue. The open PB framework has some interstitial sites and vacancies where counter-cations and other small molecules can be intercalated. During this process, Prussian blue nanocrystals act as efficient electron transport mediators between the electrode and H_2_O_2_ in solution.

### 3D porous Prussian blue nanocrystal-based cell interface

**Fig. 4:**
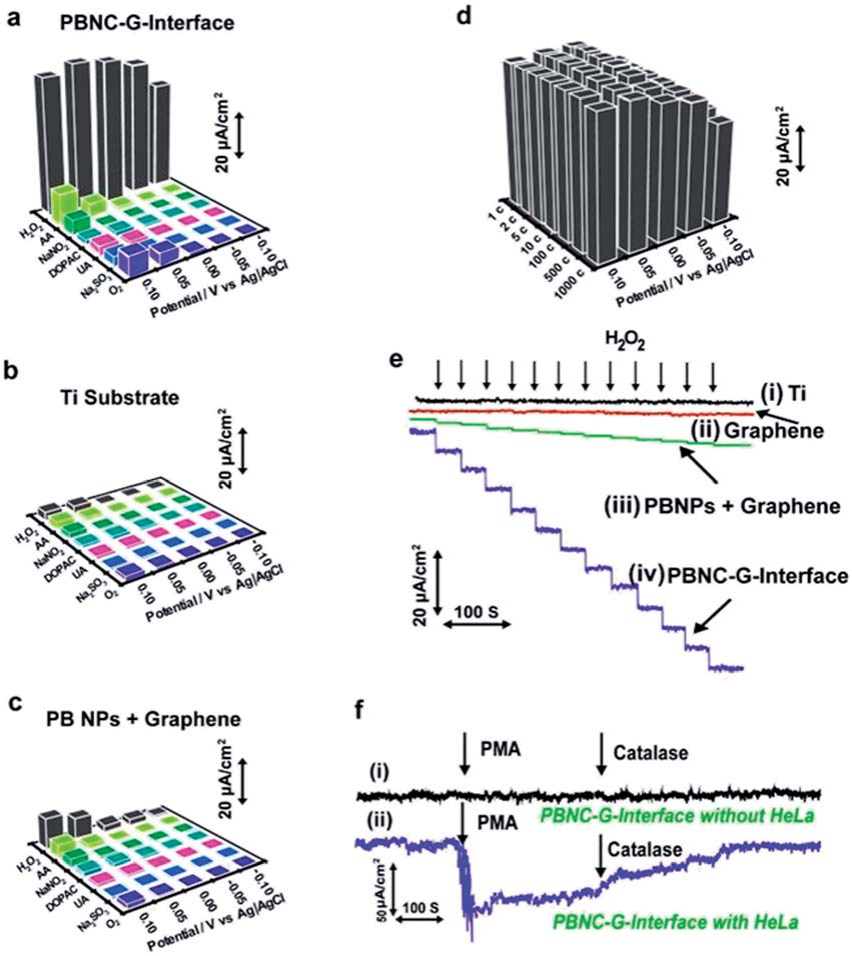
Performance of the porous Prussian blue–graphene (PBG)–cell interface. (a–c) Selectivity and sensitivity profile of the present Prussian blue nanocrystal–graphene interface (PBNC–G interface). (a) Current signals for the PBG interface obtained at different applied potentials: –0.10, –0.05, 0.00, 0.05 and 0.10 V *versus* Ag/AgCl. (b) Control experiment with black Ti substrate. (c) Control experiment with traditionally mixed PB nanoparticle–graphene (PB NPs + graphene) interface. (d) Stability of the present PBG interface. (e) Typical amperometric responses of black Ti substrate (i), black graphene without PB (ii), traditionally mixed PB nanoparticle–graphene interface (iii), and the present PBG interface (iv) to successive additions of 10 μM H_2_O_2_ at an applied potential of –0.05 V (*versus* Ag/AgCl) in PBS (50 mM, pH 6.0). (f) Amperometric responses obtained for the bare PBG interface (i) and the PBG interface with HeLa cells (ii). The measurements were performed in PBS (50 mM, pH 6.0, with 100 mM glucose) at an applied potential of –0.05 V (*versus* Ag/AgCl), after the injection of 50 mM PMA and 300 U mL^–1^ catalase.

The 3D Prussian blue–graphene frameworks were further demonstrated as direct growth interfaces for living cells. The uniform coverage of the ultrasmall porous Prussian blue nanocrystals offers an excellent substrate for cell attachment and growth, which can subsequently be used to probe different cell functions (Fig. 3e and f). The Prussian blue–graphene interface further provides a robust substrate for site-selective cell adhesion and cultivation of living cells, exhibiting high biocompatibility (>80%) and excellent biostability towards living cells (up to 120 h) (Fig. S5†). The biomonitoring performance of the Prussian blue–graphene interface for monitoring of H_2_O_2_ was extensively investigated. The amperometric responses obtained at the biointerface between the porous Prussian blue nanocrystal–graphene and HeLa cells were measured in 50 mM PBS (pH 6.0) at an applied potential of –50 mV *versus* Ag/AgCl (Fig. 4). As the interference of coexisting molecules, such as ascorbic acid, uric acid and so on, may affect the electrochemical monitoring of H_2_O_2_, the bias potential should be selected to optimize the cathodic current and sensitivity obtained at the porous Prussian blue nanocrystal–graphene electrodes. Amperometric experiments were carried out to investigate the responses of Prussian blue nanocrystal–graphene towards H_2_O_2_ at potentials of –0.10, –0.05, 0.00, 0.05, and 0.10 V (*versus* Ag/AgCl). Several interfering molecules, including O_2_, Na_2_SO_3_, uric acid (UA), 3,4-dihydroxyphenylacetic acid (DOPAC), NaNO_2_, and ascorbic acid (AA) were tested (Fig. 4a). In general, a low anodic current was obtained for these interfering molecules at relatively negative potentials. For example, the ratio of the anodic current of H_2_O_2_ and ascorbic acid (0.1 mM each) increased from 6.8 to 55 when the applied potential was reduced from 0.10 to –0.05 V (*versus* Ag/AgCl), leading to an increased selectivity.^31^ Hence, –0.05 V (*versus* Ag/AgCl) was selected as the optimized operational bias potential. In contrast, control experiments with a black Ti substrate and a traditionally mixed PB nanoparticle–graphene interface did not show similar high current signals or signal ratios even at an applied potential of –0.05 V (Fig. 4b and c), suggesting that the direct growth and attachment of ultrasmall porous PB nanocrystals on graphene enhances sensitivity and selectivity. In addition, these data confirm the biomimetic enzymatic amplification nature of H_2_O_2_ catalysis at the Prussian blue nanocrystal–graphene interface. The long-term stability of the porous Prussian blue nanocrystal–graphene interface was demonstrated by measuring repeated CV cycles at different bias voltages. The Prussian blue nanocrystal–graphene interface maintained 95% of the initial signal response even after 1000 cycles (Fig. 4d). Almost negligible current responses were observed for the black Ti substate (Fig. 4e, line i) and black graphene without PB (Fig. 4e, line ii) at the optimized potential of –50 mV. Amperometric responses of the traditionally mixed PB nanoparticle–graphene interface (Fig. 4e, line iii) and the present PBG interface (Fig. 4e, line iv) to successive additions of H_2_O_2_ were measured. Significant stepwise enhancements of the current signal correlate well with each addition of H_2_O_2_ (Fig. 4e, line iv). For the living cell interface, on the injection of 50 mM phobol 12-myristate-13-acetate (PMA) into the HeLa cell-NW assay, an increase in cathodic current was observed (Fig. 4f). No response was observed for the bare Prussian blue nanocrystal–graphene interface with the same addition of PMA (Fig. 4f, line i). However, an anodic current increase of ∼65.5 μA was obtained at 15 s (Fig. 4f, line ii).^38^ This phenomenon is attributed to the effect of PMA of inducing H_2_O_2_ production in the cells. Moreover, the injection of catalase solution (300 U mL^–1^ in PBS) led to a reduction of the current to almost background level, as catalase is known to inhibit PMA function (Fig. 4f, line ii). Accordingly, the increase in cathodic current at the Prussian blue nanocrystal–graphene interface located near the cells is ascribed to the enzymatic reduction of H_2_O_2_, which is effectively mediated by the ultrasmall porous PB nanocrystals grown on the graphene interface.

## Discussion

**Fig. 5:**
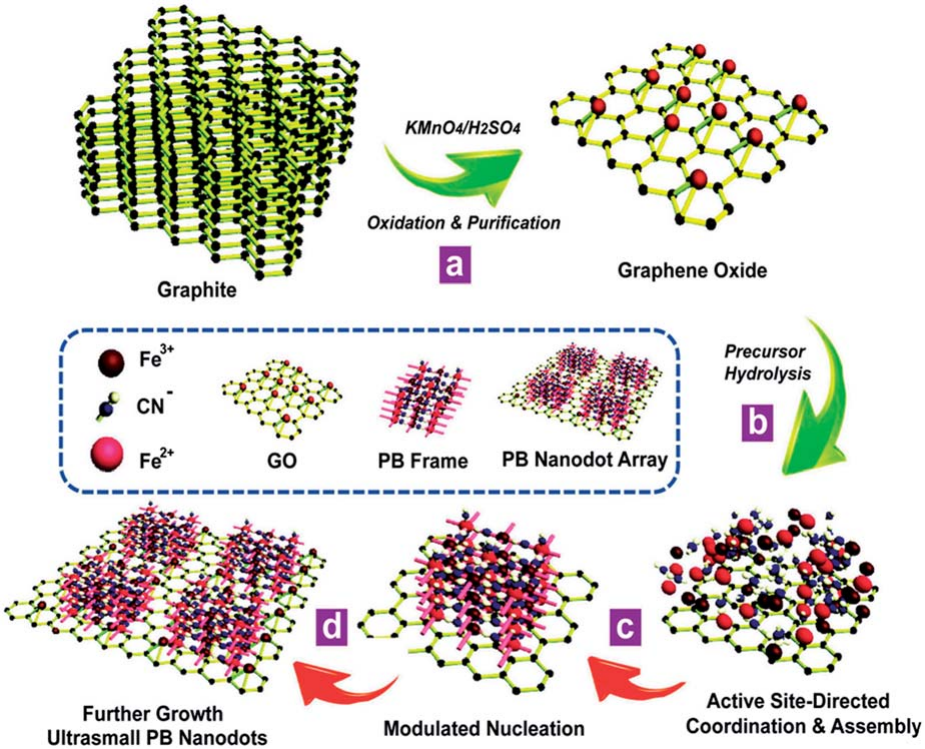
The proposed growth model for interfacial site-directed assembly of ultrasmall porous Prussian blue nanodots. (a) Graphene oxide is obtained from natural graphite by the well-known Hummers method with minor modifications. (b) The interfacial interactions of Prussian blue nanocrystal precursors with active hydrophilic sites on the graphene surface. (c and d) The *in situ* active site-directed coordination and assembly, modulated nucleation, and further growth of Prussian blue nanocrystals on graphene.

The synthetic process for the proposed *in situ* interfacial site-directed atom-level assembly on the graphene interface without capping agents is illustrated as follows (Fig. 5). The merit of this strategy is that the heterointerfaces are produced directly from GO in a wet-chemical reaction, where the *in situ* reduction of GO and growth of ultrasmall sub-5 nm porous Prussian blue nanocrystals occur simultaneously. The strong preference for interfacial site-directed assembly is due to the fastest reduction and growth occurring at the reactive sites of graphene. First, graphene oxide nanosheets are obtained from natural graphite by the well-known Hummers method with minor modifications and dispersed in DI water (Fig. 5a). Then, the *in situ* assembly of ultrasmall nanocrystals occurs at the water/graphene interface through interfacial interactions of Prussian blue nanocrystal precursors with active hydrophilic sites on the graphene surface (Fig. 5b).

The large graphene nanosheets act as excellent supports and stabilizers for the Prussian blue nanocrystals. The *in situ* active site-directed coordination and assembly, modulated nucleation, and further growth of Prussian blue nanocrystals on graphene are shown in Fig. 5c and d. As the two reactants have different coordination functionalities, they can only react by *in situ* interfacial site-directed atom-level assembly on graphene, preventing the formation of aggregates and preserving the porous, single-layer structure. This mechanism was also confirmed by two control experiments (Fig. S6 and S7†). Prussian blue on graphene was synthesised under the same conditions, except with most of the reactive sites removed from the graphene oxide (Fig. S6†) or with additional reduction to mediate the reaction rate (Fig. S7†), and in both cases no uniform ultrasmall Prussian blue nanocrystal arrays on graphene were obtained.

The porous sub-5 nm nanocrystal–graphene heterointerface possesses several important features. First, the *in situ* wet-chemical growth route provides a desirable platform for constructing heterointerfaces with improved electron transfer properties and greatly enhanced sensitivity. The enhanced sensitivity of the Prussian blue nanocrystal–graphene is attributed to the unique ultrasmall nanocrystal–graphene heterostructure, in which intimate contact between the PB nanocrystals and the graphene substrate leads to synergistic properties of both components. Second, the ultrasmall porous nanocrystals on the surface of graphene result in a high surface area, persistent catalytic activity and high site-selective cell bioaffinity. The porous PB nanocrystal–graphene interface offers a robust substrate for site-selective cell adhesion and cultivation of living cells, as the porous nanocubes exhibit high selectivity and bioaffinity toward cells, as well as excellent biostability under cell culture adhesion conditions (up to 120 h). Meanwhile, the porous heterointerfaces can also serve as long-term stable and sensitive sensing elements for H_2_O_2_, due to inherent biomimetic enzymatic activity, high surface area and ease of capturing and recognising signal molecules in 3D-space.

Compared to conventional PB electrochemical interfaces obtained from physical mixing or direct coordination, the electrocatalytic activity is enhanced at the Prussian blue nanocrystal–graphene biointerface, due to the rapid charge transport realized by the ultrathin nanostructure of the heterointerface and intimate contact between the nanocrystals and graphene. Thus, this porous Prussian blue nanocrystal–graphene interface provides a new platform for reliable and durable determination of biomolecules in living cells. Importantly, this design of heterostructures can be used for other biointerfaces to construct a series of electrochemical nanodevices, exhibiting high sensitivity and long-term stability for the monitoring of biomolecules.

## Conclusions

In summary, a site-directed, capping-agent-free growth method for porous sub-5 nm nanocrystals on graphene has been proposed. As a proof-of-concept, the ultrasmall sub-5 nm porous Prussian blue nanocrystals show narrow size distribution (4 ± 1.5 nm), high surface area (∼855 m^2^ g^–1^), fast electron transfer (rate constant of ∼9.73 s^–1^), and excellent and persistent catalytic activity (more than 450 days). Specifically, the ultrasmall porous Prussian blue nanocrystal–graphene heterointerface exhibits bio-electrochemical, synergistic and selective catalytic functionalities, allowing an unprecedented sub-nanomolar level (∼0.5 nM limit of detection) for capturing and recognition of hydrogen peroxide (H_2_O_2_) that has not yet been demonstrated with traditional biointerfaces. This approach adds to the synthetic toolbox for nanocrystals and porous materials, creating ultrasmall porous nanostructures from interfacial site-directed growth. Furthermore, the results should provide an improved understanding of the synergistic effect resulting from the integration of small size and accessible porosity, which is important for developing heterointerfaces for biocatalysis applications.

## Supplementary Material

Supplementary information
