# Supplementary material for: Sub-5 nm porous nanocrystals: interfacial site-directed growth on graphene for efficient biocatalysis
Source: Chem Sci. 2015 Apr 14;6(7):4029–34. doi: 10.1039/c5sc00819k (PMC5497271; doi:10.1039/c5sc00819k)
Supplement: Supplementary file 1 [file SC-006-C5SC00819K-s001.pdf]

Electronic Supplementary Material (ESI) for Chemical Science.
This journal is © The Royal Society of Chemistry 2015

Supporting Information

Sub-5 nm Porous Nanocrystals: Interfacial Site-Directed Growth on Graphene for Efficient Biocatalysis

Biao Kong[a,b†], Xiaotian Sun[c†], Cordelia Selomulya[b], Jing Tang[a], Gengfeng Zheng[a], Yingqing Wang[c*] and Dongyuan Zhao[a,b*]

[a] *Department of Chemistry, Collaborative Innovation Center of Chemistry for Energy Materials (iChEM), Laboratory of Advanced Materials, Shanghai Key Laboratory of Molecular Catalysis and Innovative Materials, Fudan University, Shanghai 200433, P. R. China*
[b] *Department of Chemical Engineering, Monash University, Clayton, VIC 3800, Australia*
[c] *Department of Cardiothoracic Surgery, Huashan Hospital of Fudan University, Shanghai 200040, P. R. China*

Email addresses: dyzhao@fudan.edu.cn; wangyiqing@huashan.org.cn

EXPERIMENTAL SECTION

Materials

Dulbecco's Modified Eagle's Medium (DMEM) (high glucose), Penicillin G, RPMI-1640, streptomycin, fetal calf serum (FCS), and trypsinase were purchased from GIBCO BRL (New York, USA). Ascorbic acid (AA), [3-(4,5-dimethylthiazol-2-yl)-2,5-diphenyltetrazolium bromide] (MTT), uric acid (UA), dopamine (DA), 3,4-Dihydroxy-Phenylacetic Acid (DOPAC), and Phorbol 12-myristate-3-acetate (PMA) were purchased from Sigma Chemical Co. (St. Louis, MO, USA). Hydrochloric acid (37 wt%), $K_3[Fe(CN)_6]\cdot 3H_2O$, $NaNO_2$, Na_2SO_3, and H_2O_2 were purchased from Sinopharm Chemical Reagent Co., Ltd. (China). Other reagents were analytical grade and used as purchased. All the solutions were prepared by Milli-Q water and deaerated with high-purity nitrogen before experiments.

Methods

The absorption spectra were collected using a UV-vis spectrometer (wavelength range, 190-1100 nm) (Agilent 8453, USA). The Scanning electron microscopy (SEM) investigations were carried out with a FEI Nova NanoSEM 450 instrument (FEI Company, USA) operated at 5 kV and a scanning electron microscope (HITACHI, S-4800, Japan) operated at 1 kV. The photographs for 3D sub-5 nm porous Prussian blue nanocrystals and 3D Prussian blue nanocrystal hydrogel were taken with a digital camera (Canon EOS 450D, Japan). Transmission electron microscopy (TEM) analyses were carried out with FEI TEM (Tecnai G2 T20, USA) with twin equipment operating at 200 kV. The electron diffraction X-ray spectroscopy (EDX) spectra were taken with a transmission electron microscope (JEM-2100F, Japan) at an acceleration voltage of 200 kV. Fourier transform infrared (FTIR) spectra were obtained on a Bruker Vector 22 spectrophotometer (Shimadzu, Japan) and recorded on solid samples in a KBr matrix in the range of $4000 - 500\ cm^{-1}$. The X-ray diffraction (XRD) measurement was carried out with a Bluker D8-Advance diffractometer (2θ range: 10 – 80 °) with Cu-Kα radiation (λ = 1.5418 Å). X-ray photoelectron spectra (XPS) were recorded with a Perkin-Elmer PHI 5000C ESCA system (dual X-ray source) by using Mg anode and a hemispherical energy analyzer (ultrahigh vacuum, $< 10^{-6}$ Pa, pass energy of 93.90 eV). All the energies were calibrated with contaminant carbon (C_{1s} = 284.6 eV) as a reference. Electrochemical measurements were performed on electrochemical work stations (CHI 660 and 832, CH Instruments) and Autolab General Purpose Electrochemical System (AUT20. FRA2-Autolab, Eco Chemie, B.V., The Netherlands). Cyclic voltammetry (CV) was carried out to investigate the electrochemical properties of the Prussian blue-graphene interface in a KH_2PO_4/K_2HPO_4 buffer solution (0.05 M, pH 6.0). The

reference electrode was a KCl-saturated Ag|AgCl electrode, while the auxiliary electrode was a platinum wire. All the experiments were performed at room temperature and the pH value was calibrated with a pH meter.

Fabrication of sub-5 nm porous Prussian blue nanocrystals

The porous Prussian blue nanocrystals were prepared by an interfacial site-directed, capping-agent-free growth method. First, graphene oxide (GO) was synthesized from the natural graphite powder by using a modified Hummers' method. Graphene oxide was dispersed in water by sonication, reaching a concentration up to 4.0 mg mL^{-1}. Next, 136 mg of $K_3[Fe(CN)_6]\cdot 3H_2O$ and 40 mL of hydrochloric acid (0.01 M) were slowly added to 40 mL of GO dispersion to form a stable aqueous suspension under stirring for 30 min. After that, the container was placed into an oven and heated at 85 °C for 24 h. The obtained Prussian blue-graphene composite was then separated by centrifugation for 5 min. For the 3D Prussian blue-graphene composite, these above components were hydrothermally assembled at 180 ℃ for 12 h to form a Prussian blue-graphene-based 3D hydrogel. The as-prepared hydrogel was directly dehydrated via a freeze-drying process to maintain the 3D monolithic architecture and then used as electrodes for biosensing. The final product from this process was a black monolithic hybrid aerogel composed of graphene networks and sub-5 nm Prussian blue nanocrystals. For the 3D Ti foil-supported Prussian blue-graphene electrode, a Ti foil (1.2 cm × 3.5 cm) was first put into the container of above components before hydrothermal assembly (180 ℃, 12 h) to form a Ti-Prussian blue-graphene-based electrode.

Cell culture

HEK 293T, H1299, HeLa cell lines were used in experiments. Cells were grown in Dubelcco's Modified Eagle Medium (DMEM, high glucose) supplemented with 10 % fetal bovine serum (FBS), 1 % penicillin (37 °C, 5 % CO_2), and 1 % streptomycin GIBCOBRL (Grand Island, New York, USA). The cells were lifted with trypsin-EDTA after reaching 80 – 90 % confluence and then were dispersed and diluted in DMEM (high glucose) medium. After centrifugation (1000 rpm, 5 min), the cells were re-suspended in DMEM (high glucose) medium and the cell number was counted by a hemocytometer method after removing the supernatant. Cells were then seeded into a Nunc Immuno OmniTray (Nalge Nunc International, Rochester, NY) with density of approximately 1.2×10^4 cells per square centimeters. Cells were subsequently incubated at 37 °C in a 5 % CO_2 humid incubator. The number of viable cells was determined by the 3-(4,5-dimethylthiazole-2-yl)-2,5-phenyltetrazolium bromide MTT assay. The cells cultured without biointerface culture medium were

set as controls. The absorbance was measured by testing the wavelength at 570 nm and a reference wavelength at 630 nm to obtain sample signal ($OD_{570} - OD_{630}$) *via* an ELISA plate reader using a Multiskan MK3 microplate photometer (Thermo Scientific, USA).[S1,S2]

Electrochemical kinetics at Prussian blue-graphene interface

The kinetics of hydrogen peroxide reduction at Prussian blue-graphene heterostructure was investigated using a rotating disk electrode. In irreversible electrode reactions, the current density was given by the Koutetchky-Levich equation:

$1/I = 1/I_{kin} + 1/I_L$

where I_L is the current density limited by diffusion and I_{kin} is the current density limited by electron transfer.

The Levich equation for the difiision-limited current is

$I_L = 0.62nFD^{2/3}v^{-1/6}Co\omega^{1/2}$

where I_L is the concentration of electroactive species, D is the diffusion coefficient, w is the angular velocity, n is the number of electrons transferred, F is the Faraday constant, and v is the kinematic viscosity.

The kinetic part of Koutetchky-Levich equation is obtained when the current density is extrapolated to $\omega \to \infty$

Clearly, $I_{kin} = nFkC_o$, where k is the potential-dependent rate constant of electron transfer.[S3]

Supporting Figures:

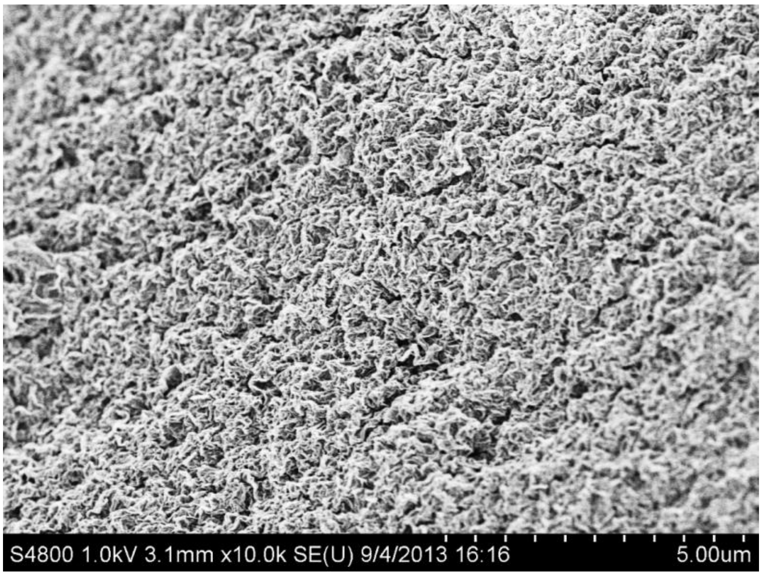

Figure S1. The SEM image of ultrasmall porous Prussian blue-graphene composite obtained by the interfacial site-directed growth method.

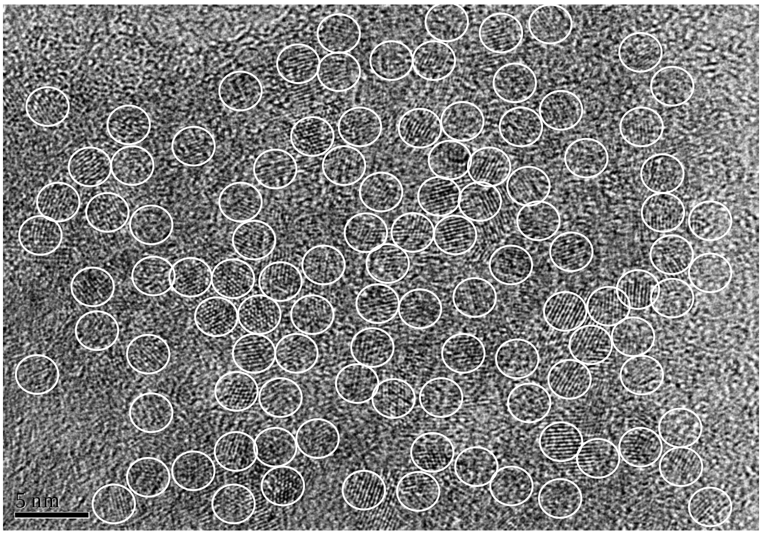

Figure S2. The HRTEM image of ultrasmall porous Prussian blue-graphene composite obtained by the interfacial site-directed growth method.

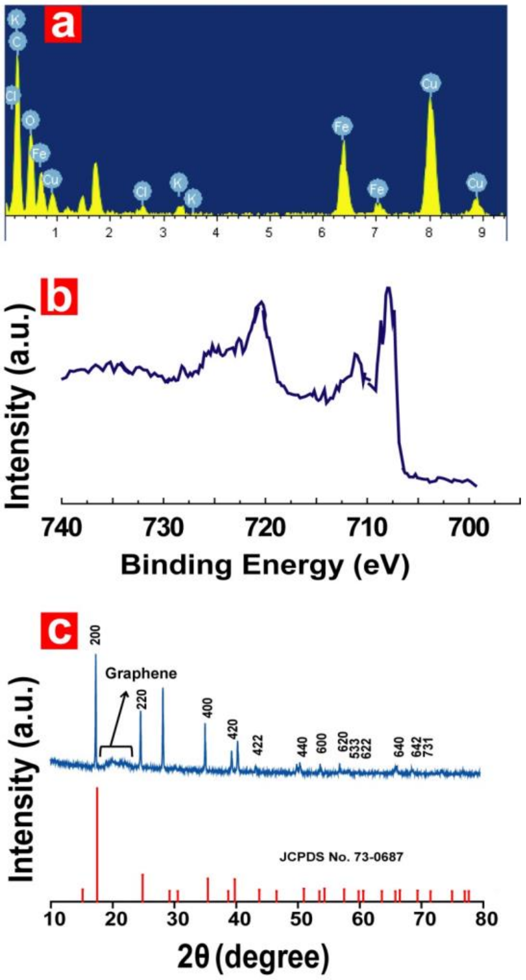

Figure S3. The EDX pattern (a), XPS spectra (b), and XRD pattern (c) of the ultrasmall porous Prussian blue-graphene (USPB-G) composite structure obtained by the interfacial site-directed growth method.

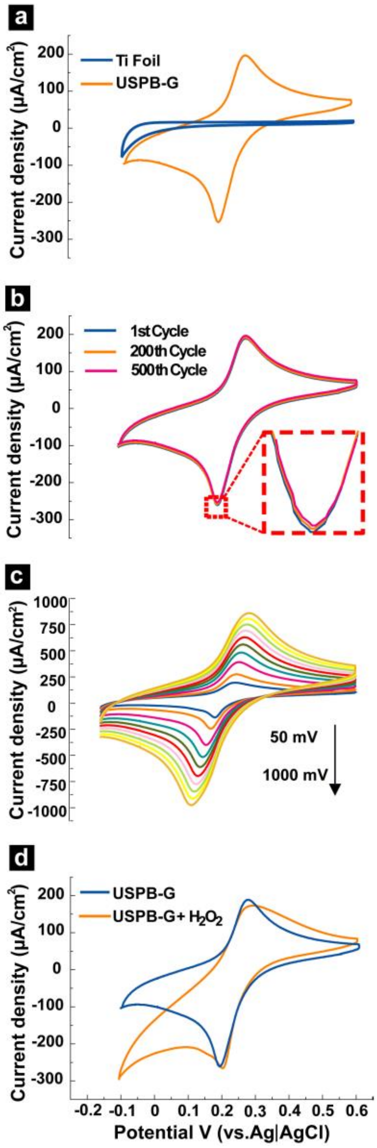

Figure S4. Electrochemical performance of the obtained ultrasmall Prussian blue-graphene composite**. (a)** Cyclic voltammograms (CVs) of the blank Ti foil (blue curve) and USPB-G (yellow curve) in N_2-saturated PBS solution (0.05 M, pH 6.0) at a scan rate of 50 mV s^{-1}. An Ag/AgCl electrode was used as a reference electrode. **(b)** The CVs of the first cycle (blue curve), 200th cycle (yellow curve), and 500th cycle (pink curve) of the USPB-G electrode in N_2-saturated PBS solution (0.05 M, pH 6.0) at a scan rate of 50 mV s^{-1}. **(c)** CVs of USPB-G electrode in N_2-saturated PBS solution (0.05 M, pH 6.0) at different scan rates: 50–1000 mV s^{-1} from the inside to the outside. **(d)** The CVs of a USPB-G electrode in N_2-saturated PBS solution (0.05 M, pH 6.0) in the absence (blue curve) and presence of 5 mM H_2O_2 (yellow curve) at a scan rate of 50 mVs^{-1}.

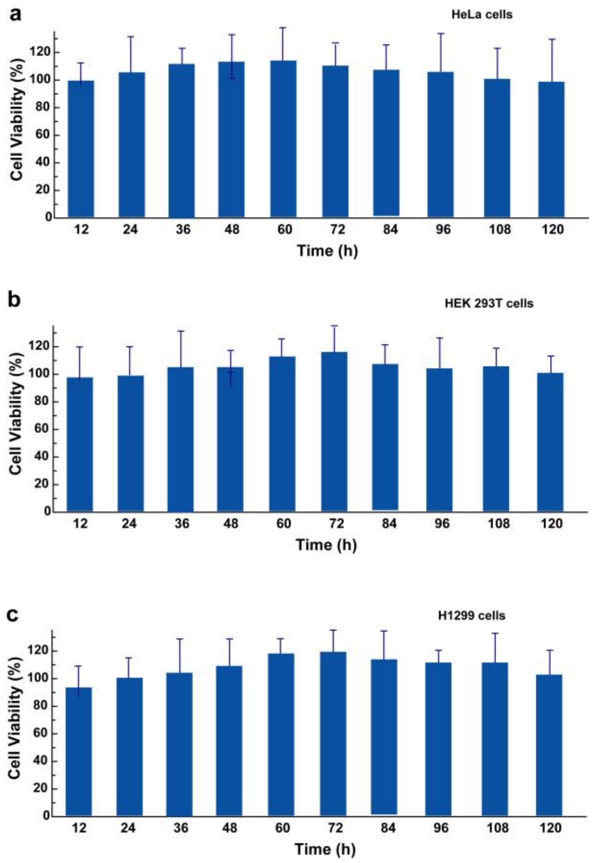

Figure S5. Interface growth inhibition data for HeLa, HEK 293T, H1299 cells treated with various growth interfaces of Prussian blue-graphene after the incubation for 12-120 h.

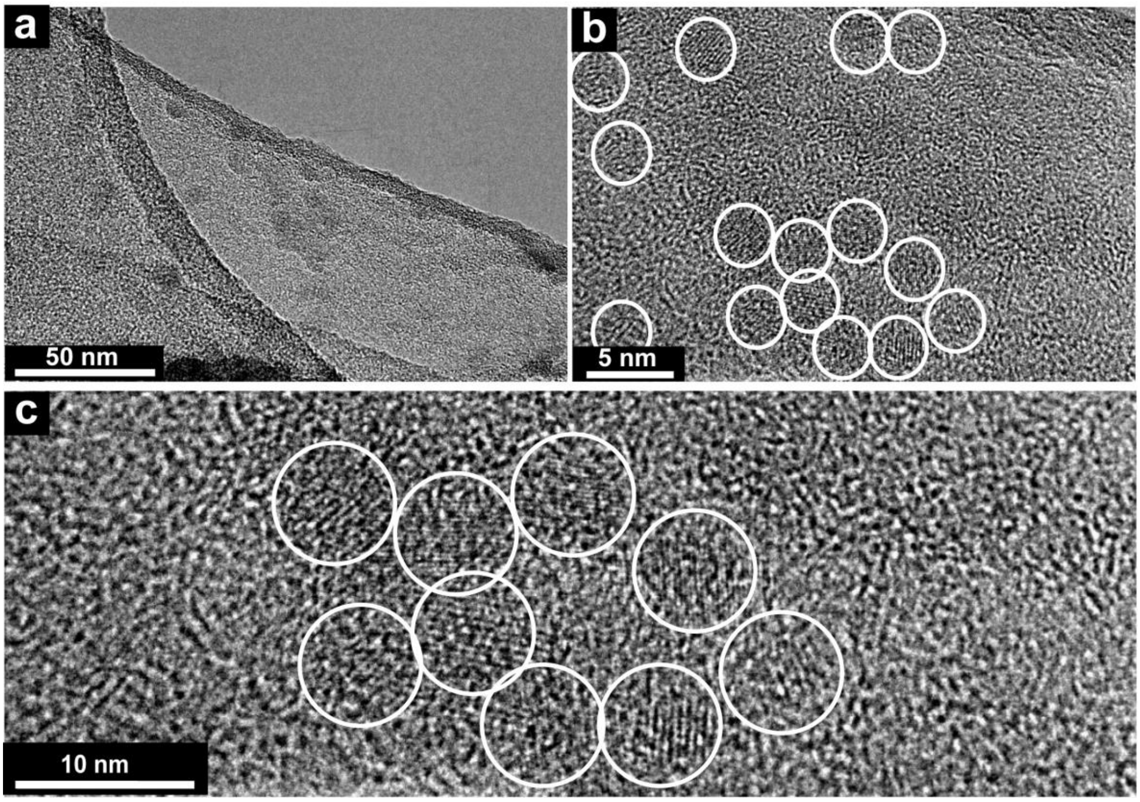

Figure S6. TEM(a, b) and HRTEM (c) images of the Prussian blue on interfacial site-free graphene as a control experiment. The Prussian blue was obtained by the same synthesis condition with the obtained ultrasmall porous Prussian blue-graphene except the graphene oxide was reduced for 1 h at 100 °C in 55% hydroiodic (HI) acid to remove the most reactive sites.

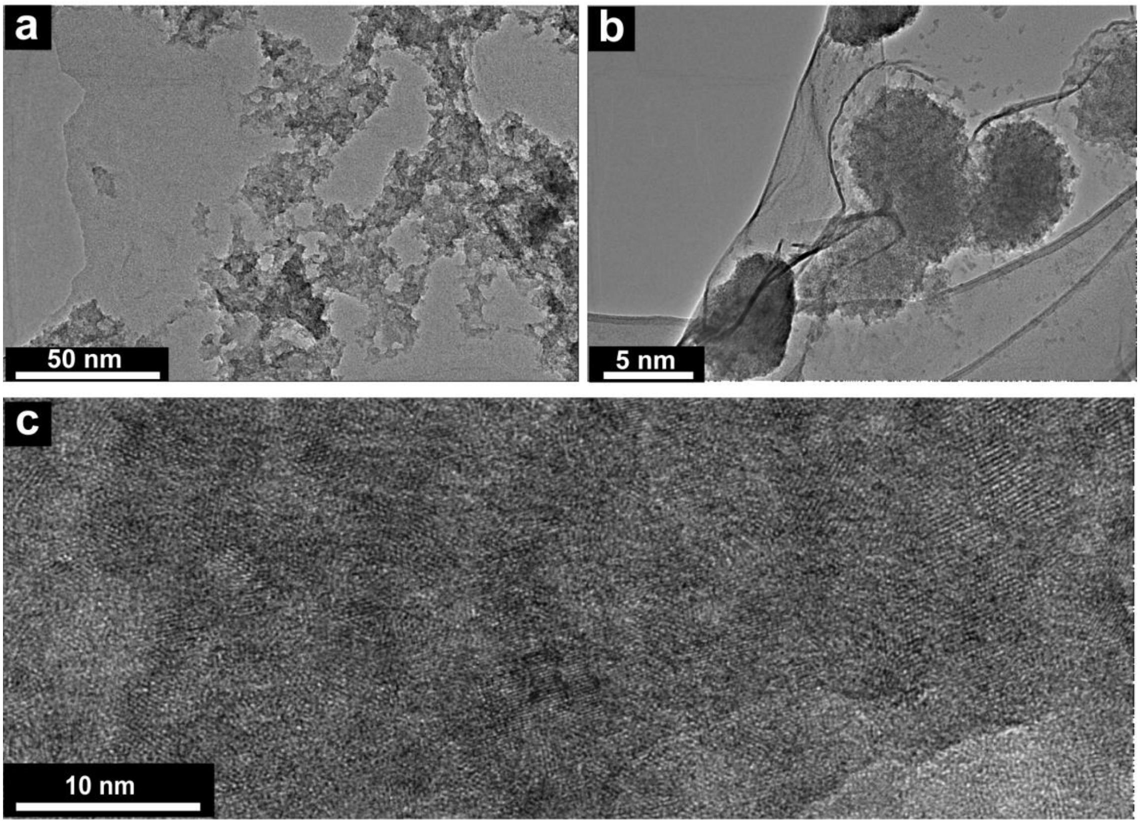

Figure S7. The TEM (a, b) and HRTEM (c) images of obtained Prussian blue on graphene, triggered by exogenous oxidant as a control experiment. The Prussian blue was obtained by the same synthesis condition with the obtained ultrasmall porous Prussian blue-graphene except the adding of reduction to mediate the reaction rate.

Table S1. **Analytical performance of the present 3D porous Prussian blue-graphene (PBG)-based real time recognition and recording of H_2O_2, compared to previously reported literatures**

Nanoelectrode Biointerfaces	*AP(mV)*	*LR (µm)*	*LOD (µm)*	*Reference*
3D Prussian blue-graphene	-50 (0.0 V vs Ag|AgCl)	0.01-2.0×10^4	0.0005	*a*
Cyt c/TiO$_2$ nanoneedles	-45 (0.0 V vs Ag|AgCl)	0.85-2.4×10^4	94.6	*S2*
Cyt c/Nanoporous Au	-100 (0.0 V vs Ag|AgCl)	10-1.2×10^4	6.3	*S3*
Cyt c/Au-NR	-100 (0.0 V vs Ag|AgCl)	50-1.5×10^3	3.7	*S4*
Cyt c/Au/CP	-100 (0.0 V vs Ag|AgCl)	10-1.0×10^3	10	*S5*
Cyt c/Au/Chit	-250 (0.0 V vs Ag|AgCl)	8.5×10^2-1.3×10^4	9.8	*S6*
HRP/Clay/Chit/Au	-300 (0.0 V vs SCE)	39-3.1×10^3	9	*S7*
HRP/Au/TiO$_2$	-600 (0.0 V vs Ag|AgCl)	5-4×10^2	2	*S8*
Hb/CMC-TiO$_2$ nanotubes	-300 (0.0 V vs SCE)	4-64	4.637	*S9*
Mb/titanate nanotubes	~ -290 (0.0 V vs SCE)	2-160	0.6	*S10*
Mb/titanate nanosheets	~ -310 (0.0 V vs SCE)	2-160	0.6	*S11*
HRP/TiO$_2$ nanoparticles	0 (0.0 V vs SCE)	7.5-123	2.5	*S12*
HRP/Th-TiO$_2$ nanotubes	-645 (0.0 V vs SCE)	10-3.0×103	-	*S13*
HRP-TiO$_2$ sol-gel	-250 (0.0 V vs SCE)	4-1.0×10^3	0.8	*S14*
Biomimetic TiO$_2$-PB NWs	-50 (0.0 V vs Ag|AgCl)	0.01-5.0×10^4	0.02	*S1*
MnO NP@Mesoporous Carbon	600-700 (0.0 V vs Ag|AgCl)	2 µM–2.4 mM	2	*S15*

[a] The present work.

Applied Potential = AP; Linear Range = LR; Limit of Detection = LOD;

Cyt *c* = Cytochrome c; Hb = Hemoglobin; Mb = Myoglobin; HRP = Horse Radish Peroxidase;

Th = thionine chloride; Chit = chitosan; PB = Prussian blue; NP = Nanoparticle

References:

(S1) B. Kong, J. Tang, Z. Wu, C. Selomulya, H. Wang, J. Wei, Y. Wang, G. Zheng and D. Zhao, *NPG Asia Mater*. 2014, **6**, e117

(S2) J. Tang, Y. Zhang, B. Kong, Y. Wang, P. Da, J. Li, A. A. Elzatahry, D. Zhao, X. Gong, G. Zheng, *Nano Lett.*, 2014, **14**, 2702.

(S3) A. A. Karyakin, O. V. Gitelmacher, E. E. Karyakina, *Anal. Chem.*, 1995, **67**, 2419.

(S2) Y. Luo, H. Liu, Q. Rui, Y. Tian, Y. *Anal. Chem.*, 2009, **81**, 3035.

(S3) A. Kafi, G. Wu, A. Chen, *Biosens. Bioelectron.*, 2008, **24**, 566.

(S4) A. Zhu, Y. Tian, H. Liu, Y. Luo, *Biomaterials*, 2009, **3**0, 3183.

(S5) H. Liu, Y. Tian, P. Xia, *Langmuir*, 2008, **24**, 6359.

(S6) H. Ju, S. Liu, B. Ge, F. Lisdat, F. Scheller, *Electroanalysis*, 2002, **14**, 141.

(S7) J. Feng, G. Zhao, J. Xu, H. Chen, *Anal. Biochem.*, 2005, **342**, 280.

(S8) X. Zhao, Z. Mai, X. Kang, X. Zou, *Biosens. Bioelectron.*, 2008, **23**, 1032.

(S9) W. Zheng, Y. Zheng, K. Jin, N. Wang, *Talanta*,2008, **74**, 1414.

(S10) A. Liu, M. Wei, I. Honma, H. Zhou, *Anal. Chem.*, 2005, **77**, 8068.

(S11) L. Zhang, Q. Zhang, J. Li, *Adv. Funct. Mater.*, 2007, **17**, 1958.

(S12) Y. Zhang, P. He, N. Hu, *Electrochim. Acta*, 2004, **49**, 1981.

(S13) P. Xiao, B. B. Garcia, Q. Guo, D. Liu, G. Cao, *Electrochem. Commun.*, 2007, **9**, 2441.

(S14) H. Lu, J. Yang, J. F. Rusling, N. Hu, *Electroanalysis*, 2006, **18**, 379.

(S15) T. Wang, Z. Peng, Y. Wang, J. Tang, G. Zheng, *Sci.Rep.*, 2013, **3**, 2693.
